# (R*,S*)-(±)-1-(2-{[2,8-Bis(trifluoromethyl)quinolin-4-yl](hydroxy)methyl}piperidin-1-yl)ethanone methanol monosolvate

**DOI:** 10.1107/S1600536811038128

**Published:** 2011-09-30

**Authors:** Raoni S. B. Gonçalves, Marcus V. N. de Souza, Solange M. S. V. Wardell, James L. Wardell, Edward R. T. Tiekink

**Affiliations:** a FioCruz-Fundação Oswaldo Cruz, Instituto de Tecnologia em Fármacos-Far Manguinhos, Rua Sizenando Nabuco, 100, Manguinhos, 21041-250, Rio de Janeiro, RJ, Brazil; b CHEMSOL, 1 Harcourt Road, Aberdeen AB15 5NY, Scotland; c Centro de Desenvolvimento Tecnológico em Saúde (CDTS), Fundação Oswaldo Cruz (FIOCRUZ), Casa Amarela, Campus de Manguinhos, Av. Brasil 4365, 21040-900, Rio de Janeiro, RJ, Brazil; d Department of Chemistry, University of Malaya, 50603 Kuala Lumpur, Malaysia

## Abstract

The title mefloquine derivative has been crystallized as its 1:1 methanol solvate, C_19_H_18_F_6_N_2_O_2_·CH_3_OH. Each of the meth­ine­hydroxyl residue [the C—C—C—O torsion angle is −16.35 (17) °] and the piperidinyl group [distorted chair conformation] lies to one side of the quinolinyl ring system. The hydroxyl and carbonyl groups lie to either side of the mol­ecule, enabling their participation in inter­molecular inter­actions. Thus, the hydroxyl and carbonyl groups of two centrosymmetrically related mol­ecules are bridged by two methanol mol­ecules *via* O—H⋯O hydrogen bonds, leading to a four-mol­ecule aggregate. These are linked into a supra­molecular chain along the *a* axis *via* C—H⋯O inter­actions involving the hydroxyl-O atom. The chains assemble into layers that inter­digitate along the *c* axis being connected by C—H⋯F inter­actions.

## Related literature

For background to the use of quinoline derivatives, including mefloquine derivatives, for the treatment of tuberculosis, see: de Souza *et al.* (2009 ▶); Candea *et al.* (2009 ▶); Danelishvili *et al.* (2005 ▶); Kunin & Ellis (2008 ▶); Jayaprakash *et al.* (2006 ▶); Bermudez *et al.* (2004 ▶). For related structural studies of mefloquine derivatives, see: Wardell *et al.* (2010 ▶, 2011 ▶).
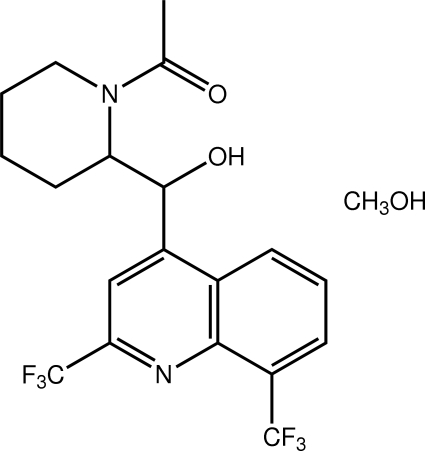


## Experimental

### 

#### Crystal data

C_19_H_18_F_6_N_2_O_2_·CH_4_O
*M* *_r_* = 452.40
Triclinic, 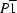

*a* = 9.4719 (2) Å
*b* = 10.1223 (3) Å
*c* = 11.9227 (3) Å
α = 114.567 (1)°
β = 90.343 (2)°
γ = 102.795 (2)°
*V* = 1007.61 (4) Å^3^
*Z* = 2
Mo *K*α radiation
μ = 0.14 mm^−1^
*T* = 120 K
0.20 × 0.08 × 0.08 mm

#### Data collection

Nonius KappaCCD diffractometer
Absorption correction: multi-scan (*SADABS*; Sheldrick, 2007 ▶) *T*_min_ = 0.883, *T*_max_ = 1.000
20055 measured reflections
4602 independent reflections
4038 reflections with *I* > 2σ(*I*)
*R*_int_ = 0.041

#### Refinement

*R*[*F* ^2^ > 2σ(*F* ^2^)] = 0.040
*wR*(*F* ^2^) = 0.110
*S* = 1.02
4602 reflections
288 parameters
2 restraints
H atoms treated by a mixture of independent and constrained refinement
Δρ_max_ = 0.37 e Å^−3^
Δρ_min_ = −0.33 e Å^−3^

### 

Data collection: *COLLECT* (Hooft, 1998 ▶); cell refinement: *DENZO* (Otwinowski & Minor, 1997 ▶) and *COLLECT*; data reduction: *DENZO* and *COLLECT*; program(s) used to solve structure: *SHELXS97* (Sheldrick, 2008 ▶); program(s) used to refine structure: *SHELXL97* (Sheldrick, 2008 ▶); molecular graphics: *ORTEP-3* (Farrugia, 1997 ▶) and *DIAMOND* (Brandenburg, 2006 ▶); software used to prepare material for publication: *publCIF* (Westrip, 2010 ▶).

## Supplementary Material

Crystal structure: contains datablock(s) global, I. DOI: 10.1107/S1600536811038128/hb6409sup1.cif

Structure factors: contains datablock(s) I. DOI: 10.1107/S1600536811038128/hb6409Isup2.hkl

Supplementary material file. DOI: 10.1107/S1600536811038128/hb6409Isup3.cml

Additional supplementary materials:  crystallographic information; 3D view; checkCIF report

## Figures and Tables

**Table 1:** Hydrogen-bond geometry (Å, °)

*D*—H⋯*A*	*D*—H	H⋯*A*	*D*⋯*A*	*D*—H⋯*A*
O1—H1*o*⋯O3^i^	0.84 (2)	1.87 (2)	2.7121 (18)	177 (2)
O3—H3*o*⋯O2^ii^	0.85 (2)	1.83 (2)	2.6667 (17)	168 (2)
C7—H7⋯O1^iii^	0.95	2.49	3.3280 (18)	147
C17—H17*a*⋯F6^iv^	0.99	2.51	3.3123 (17)	138

Symmetry codes: (i) 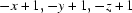; (ii) 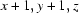; (iii) 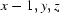; (iv) 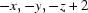.

**Fig. 1.**
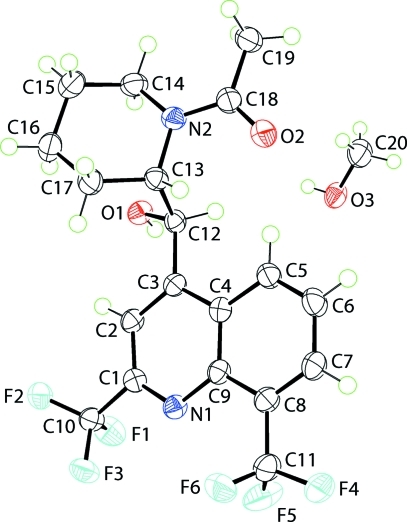
The molecular structure of (I) showing displacement ellipsoids at the 50% probability level.

**Fig. 2.**
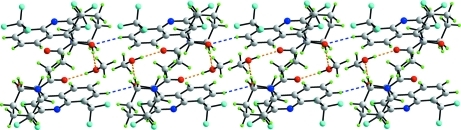
A view of a supramolecular chain in (I) aligned along the a axis. The O—H···O and C—H···O interactions are shown as orange and blue dashed lines, respectively.

**Fig. 3.**
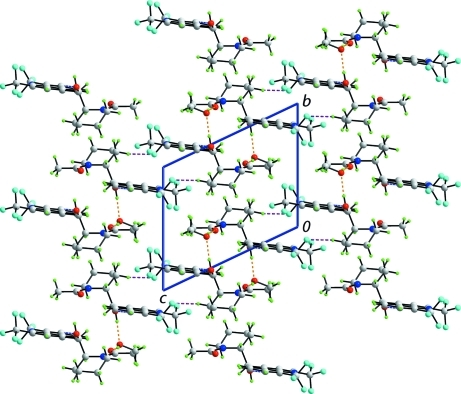
A view in projection down the a axis of the unit-cell contents in (I) highlighting the stacking of layers along c. The O—H···O, C—H···O and C—H···F interactions are shown as orange, blue and purple dashed lines, respectively.

## supplementary crystallographic information

### Comment

Tuberculosis (TB) is considered a global health emergency by the World Health Organization (WHO). Quinoline derivatives have been reported to exhibit substantial anti-mycobacterial activities and can be considered a promising area for the discovery of new anti-TB agents (de Souza *et al.*, 2009; Candea *et al.*, 2009). The quinoline derivative, mefloquine, ((*R**, S*)-(±)-α-2-piperidinyl-2,8-bis(trifluoromethyl)-4-quinolinemethanol, which has been used for a long time as an anti-malarial drug, has recently received considerable attention as an anti-mycobacterial drug. This substance has been found to possess substantial activities against Gram-positive bacteria (Kunin & Ellis, 2008) and Mycobacterium species (Danelishvili *et al.*, 2005; Jayaprakash *et al.*, 2006; Bermudez *et al.*, 2004). However, there remains a need for more active and more resistant compounds. With this in mind, the acetoamido derivative of mefloquine, (*R**, S*)-(±)-α-2-*N*-acetopiperidinyl-2,8-bis (trifluoromethyl)-4-quinolinemethanol, (I), has been prepared in continuation with biological and structural studies (Wardell *et al.*, 2010; Wardell *et al.*, 2011). Herein, we report its crystal structure.

In (I), Fig. 1, the asymmetric unit comprises a neutral mefloquine derivative and a methanol molecule of solvation. In the organic molecule, the methine-hydroxyl group is twisted out the least-squares plane through the quinolinyl ring (r.m.s. deviation = 0.008 Å) to which it is attached as seen in the value of the C2—C3—C12—O1 torsion angle of -16.35 (17) °. The piperidinyl group, with a distorted chair conformation, lies to one side and is directed away from the quinolinyl residue. Within the molecule, the hydroxyl and carbonyl groups are directed away from each other allowing for their participation in intermolecular hydrogen bonding interactions.

The formation of a centrosymmetric four molecule aggregate mediated by O—H···O hydrogen bonding, Table 1, is the most notable feature of the crystal packing. The hydroxyl group forms a donor O—H···O hydrogen bond with the solvent methanol molecule which in turn forms a O—H···O hydrogen bond with the carbonyl-O2 atom of a symmetry related molecule. In this way a centrosymmetric 18-membered {···OCNC_2_OH···OH···}_2_ synthon is formed. The four-molecule aggregates are linked into a linear supramolecular chain along the *a*-direction *via* C—H···O interactions where the acceptor atom is the mefloquine-hydroxyl group, Table 1 and Fig. 2. Chains assemble into layers in the *ab* plane and inter-digitate along the *c* axis, enabling the formation of C—H···F interactions, Table 1 and Fig. 3.

### Experimental

To a stirred solution of mefloquine (3.0 mmol) and triethylamine (7.5 mmol) in anhydrous THF (5 ml), acetyl chloride (6 mmol) was added drop wise at 273 K. The mixture stirred at room temperature for 2 h and after complete conversion of the starting material, as indicated by TLC, THF was evaporated under reduced pressure. The residue was dissolved in CH_2_Cl_2_ and washed with water (3 *x* 10 ml). The organic layer was separated, dried over anhydrous MgSO_4_, filtered, and solvent was evaporated under reduced pressure to give the desired product, which was recrystallized from MeOH as colourless blocks. *M*.pt. 458–460 K. IR ν_max_ (cm^-1^; KBr pellets): 1682 (NC═O); 1189, 1150, 1115 (C—F).

### Refinement

The C-bound H atoms were geometrically placed (C–H = 0.95–1.00 Å) and refined as riding with *U*_iso_(H) = 1.2–1.5*U*_eq_(C). The O-bound H atoms were located from a difference map and their positions refined with O—H = 0.84±0.01 Å, and with *U*_iso_(H) = 1.5*U*_eq_(O).

### Crystal data

**Table:**

C_19_H_18_F_6_N_2_O_2_·CH_4_O	*Z* = 2
*M**_r_* = 452.40	*F*(000) = 468
Triclinic, *P*1	*D*_x_ = 1.491 Mg m^−^^3^
Hall symbol: -P 1	Mo *K*α radiation, λ = 0.71073 Å
*a* = 9.4719 (2) Å	Cell parameters from 16977 reflections
*b* = 10.1223 (3) Å	θ = 2.9–27.5°
*c* = 11.9227 (3) Å	µ = 0.14 mm^−^^1^
α = 114.567 (1)°	*T* = 120 K
β = 90.343 (2)°	Block, colourless
γ = 102.795 (2)°	0.20 × 0.08 × 0.08 mm
*V* = 1007.61 (4) Å^3^	

### Data collection

**Table:**

Nonius KappaCCD diffractometer	4602 independent reflections
Radiation source: Enraf Nonius FR591 rotating anode	4038 reflections with *I* > 2σ(*I*)
10 cm confocal mirrors	*R*_int_ = 0.041
Detector resolution: 9.091 pixels mm^-1^	θ_max_ = 27.5°, θ_min_ = 3.1°
φ and ω scans	*h* = −11→12
Absorption correction: multi-scan (*SADABS*; Sheldrick, 2007)	*k* = −13→13
*T*_min_ = 0.883, *T*_max_ = 1.000	*l* = −15→15
20055 measured reflections	

### Refinement

**Table:**

Refinement on *F*^2^	Primary atom site location: structure-invariant direct methods
Least-squares matrix: full	Secondary atom site location: difference Fourier map
*R*[*F*^2^ > 2σ(*F*^2^)] = 0.040	Hydrogen site location: inferred from neighbouring sites
*wR*(*F*^2^) = 0.110	H atoms treated by a mixture of independent and constrained refinement
*S* = 1.02	*w* = 1/[σ^2^(*F*_o_^2^) + (0.0544*P*)^2^ + 0.5339*P*] where *P* = (*F*_o_^2^ + 2*F*_c_^2^)/3
4602 reflections	(Δ/σ)_max_ = 0.001
288 parameters	Δρ_max_ = 0.37 e Å^−^^3^
2 restraints	Δρ_min_ = −0.33 e Å^−^^3^

### Special details

**Table:**

Geometry. All s.u.'s (except the s.u. in the dihedral angle between two l.s. planes) are estimated using the full covariance matrix. The cell s.u.'s are taken into account individually in the estimation of s.u.'s in distances, angles and torsion angles; correlations between s.u.'s in cell parameters are only used when they are defined by crystal symmetry. An approximate (isotropic) treatment of cell s.u.'s is used for estimating s.u.'s involving l.s. planes.
Refinement. Refinement of *F*^2^ against ALL reflections. The weighted *R*-factor *wR* and goodness of fit *S* are based on *F*^2^, conventional *R*-factors *R* are based on *F*, with *F* set to zero for negative *F*^2^. The threshold expression of *F*^2^ > 2σ(*F*^2^) is used only for calculating *R*-factors(gt) *etc*. and is not relevant to the choice of reflections for refinement. *R*-factors based on *F*^2^ are statistically about twice as large as those based on *F*, and *R*- factors based on ALL data will be even larger.

### Fractional atomic coordinates and isotropic or equivalent isotropic displacement parameters (Å^2^)

**Table:**

	*x*	*y*	*z*	*U*_iso_*/*U*_eq_	
F1	0.26612 (10)	0.38078 (10)	1.09873 (8)	0.0321 (2)	
F2	0.29759 (9)	0.17772 (11)	1.10061 (8)	0.0292 (2)	
F3	0.12314 (9)	0.27028 (11)	1.18989 (8)	0.0287 (2)	
F4	−0.46418 (10)	0.20984 (12)	0.96873 (9)	0.0365 (2)	
F5	−0.24743 (11)	0.34198 (11)	1.04600 (10)	0.0418 (3)	
F6	−0.32894 (11)	0.13779 (12)	1.06582 (8)	0.0366 (2)	
O1	0.29182 (10)	−0.01823 (11)	0.64457 (9)	0.0208 (2)	
H1o	0.315 (2)	0.0724 (12)	0.6576 (18)	0.031*	
O2	−0.10074 (12)	−0.28747 (13)	0.38799 (10)	0.0305 (3)	
N1	−0.03724 (12)	0.17012 (13)	0.97451 (11)	0.0197 (2)	
N2	0.11869 (12)	−0.30509 (13)	0.44422 (11)	0.0204 (2)	
C1	0.09757 (14)	0.15791 (15)	0.96899 (12)	0.0187 (3)	
C2	0.15919 (14)	0.08145 (15)	0.86058 (13)	0.0193 (3)	
H2	0.2584	0.0780	0.8646	0.023*	
C3	0.07291 (14)	0.01209 (14)	0.74906 (12)	0.0178 (3)	
C4	−0.07565 (14)	0.02027 (15)	0.74941 (13)	0.0187 (3)	
C5	−0.17571 (15)	−0.04706 (16)	0.63970 (13)	0.0218 (3)	
H5	−0.1441	−0.1013	0.5620	0.026*	
C6	−0.31640 (16)	−0.03461 (17)	0.64485 (14)	0.0256 (3)	
H6	−0.3819	−0.0814	0.5709	0.031*	
C7	−0.36548 (15)	0.04705 (17)	0.75876 (14)	0.0244 (3)	
H7	−0.4633	0.0558	0.7610	0.029*	
C8	−0.27259 (15)	0.11371 (16)	0.86618 (13)	0.0215 (3)	
C9	−0.12550 (14)	0.10137 (15)	0.86463 (12)	0.0188 (3)	
C10	0.19590 (14)	0.24533 (16)	1.09029 (13)	0.0214 (3)	
C11	−0.32713 (16)	0.20071 (18)	0.98679 (14)	0.0273 (3)	
C12	0.13776 (14)	−0.06447 (15)	0.62862 (12)	0.0181 (3)	
H12	0.1016	−0.0346	0.5658	0.022*	
C13	0.09029 (15)	−0.23709 (15)	0.57593 (12)	0.0193 (3)	
H13	−0.0178	−0.2641	0.5764	0.023*	
C14	0.26389 (16)	−0.33204 (17)	0.41659 (14)	0.0250 (3)	
H14A	0.3357	−0.2349	0.4399	0.030*	
H14B	0.2618	−0.3924	0.3263	0.030*	
C15	0.31159 (18)	−0.41381 (18)	0.48644 (15)	0.0299 (3)	
H15A	0.2465	−0.5157	0.4563	0.036*	
H15B	0.4119	−0.4240	0.4702	0.036*	
C16	0.30687 (17)	−0.32879 (17)	0.62526 (14)	0.0274 (3)	
H16A	0.3781	−0.2301	0.6570	0.033*	
H16B	0.3336	−0.3858	0.6689	0.033*	
C17	0.15401 (15)	−0.30642 (16)	0.65061 (13)	0.0230 (3)	
H17A	0.1562	−0.2415	0.7400	0.028*	
H17B	0.0876	−0.4049	0.6329	0.028*	
C18	0.01477 (15)	−0.32362 (15)	0.35744 (13)	0.0225 (3)	
C19	0.03672 (18)	−0.39287 (17)	0.22186 (13)	0.0275 (3)	
H19A	0.0302	−0.4999	0.1944	0.041*	
H19B	0.1329	−0.3435	0.2098	0.041*	
H19C	−0.0387	−0.3802	0.1732	0.041*	
O3	0.63629 (12)	0.72872 (12)	0.32218 (11)	0.0306 (3)	
H3O	0.7165 (15)	0.711 (2)	0.335 (2)	0.046*	
C20	0.56930 (19)	0.61623 (19)	0.20456 (17)	0.0381 (4)	
H20A	0.4786	0.6367	0.1839	0.057*	
H20B	0.5477	0.5182	0.2069	0.057*	
H20C	0.6353	0.6160	0.1415	0.057*	

### Atomic displacement parameters (Å^2^)

**Table:**

	*U*^11^	*U*^22^	*U*^33^	*U*^12^	*U*^13^	*U*^23^
F1	0.0328 (5)	0.0246 (4)	0.0284 (5)	−0.0055 (4)	−0.0033 (4)	0.0077 (4)
F2	0.0226 (4)	0.0386 (5)	0.0242 (4)	0.0117 (4)	−0.0019 (3)	0.0092 (4)
F3	0.0241 (4)	0.0398 (5)	0.0185 (4)	0.0074 (4)	0.0042 (3)	0.0090 (4)
F4	0.0211 (4)	0.0556 (6)	0.0317 (5)	0.0187 (4)	0.0061 (4)	0.0130 (5)
F5	0.0297 (5)	0.0309 (5)	0.0469 (6)	0.0096 (4)	0.0068 (4)	−0.0015 (4)
F6	0.0364 (5)	0.0529 (6)	0.0231 (5)	0.0173 (5)	0.0071 (4)	0.0155 (4)
O1	0.0163 (5)	0.0197 (5)	0.0242 (5)	0.0033 (4)	0.0029 (4)	0.0078 (4)
O2	0.0276 (5)	0.0359 (6)	0.0237 (5)	0.0124 (5)	−0.0031 (4)	0.0064 (5)
N1	0.0170 (5)	0.0196 (5)	0.0210 (6)	0.0038 (4)	0.0015 (4)	0.0077 (5)
N2	0.0205 (6)	0.0193 (5)	0.0187 (6)	0.0046 (4)	0.0010 (4)	0.0056 (5)
C1	0.0185 (6)	0.0175 (6)	0.0194 (6)	0.0027 (5)	0.0010 (5)	0.0080 (5)
C2	0.0148 (6)	0.0200 (6)	0.0222 (7)	0.0038 (5)	0.0017 (5)	0.0084 (5)
C3	0.0180 (6)	0.0156 (6)	0.0201 (6)	0.0038 (5)	0.0024 (5)	0.0080 (5)
C4	0.0173 (6)	0.0180 (6)	0.0217 (6)	0.0033 (5)	0.0012 (5)	0.0099 (5)
C5	0.0207 (7)	0.0246 (7)	0.0200 (6)	0.0057 (5)	0.0014 (5)	0.0095 (6)
C6	0.0204 (7)	0.0316 (8)	0.0226 (7)	0.0046 (6)	−0.0022 (5)	0.0104 (6)
C7	0.0160 (6)	0.0305 (7)	0.0272 (7)	0.0072 (5)	0.0019 (5)	0.0121 (6)
C8	0.0177 (6)	0.0241 (7)	0.0230 (7)	0.0056 (5)	0.0039 (5)	0.0102 (6)
C9	0.0170 (6)	0.0180 (6)	0.0213 (6)	0.0039 (5)	0.0014 (5)	0.0085 (5)
C10	0.0167 (6)	0.0244 (7)	0.0207 (7)	0.0039 (5)	0.0024 (5)	0.0079 (6)
C11	0.0180 (7)	0.0333 (8)	0.0274 (7)	0.0087 (6)	0.0021 (5)	0.0087 (6)
C12	0.0152 (6)	0.0201 (6)	0.0186 (6)	0.0035 (5)	0.0005 (5)	0.0083 (5)
C13	0.0184 (6)	0.0196 (6)	0.0183 (6)	0.0035 (5)	0.0011 (5)	0.0072 (5)
C14	0.0232 (7)	0.0262 (7)	0.0223 (7)	0.0084 (5)	0.0044 (5)	0.0060 (6)
C15	0.0302 (8)	0.0278 (8)	0.0320 (8)	0.0138 (6)	0.0027 (6)	0.0097 (6)
C16	0.0277 (7)	0.0275 (7)	0.0297 (8)	0.0109 (6)	−0.0002 (6)	0.0128 (6)
C17	0.0256 (7)	0.0207 (6)	0.0234 (7)	0.0050 (5)	0.0012 (5)	0.0103 (6)
C18	0.0257 (7)	0.0172 (6)	0.0227 (7)	0.0034 (5)	−0.0015 (5)	0.0077 (5)
C19	0.0373 (8)	0.0231 (7)	0.0206 (7)	0.0080 (6)	0.0000 (6)	0.0077 (6)
O3	0.0239 (5)	0.0236 (5)	0.0383 (6)	0.0042 (4)	−0.0052 (4)	0.0085 (5)
C20	0.0317 (9)	0.0300 (8)	0.0434 (10)	0.0044 (7)	−0.0090 (7)	0.0087 (7)

### Geometric parameters (Å, °)

**Table:**

F1—C10	1.3465 (17)	C7—H7	0.9500
F2—C10	1.3311 (16)	C8—C9	1.4257 (18)
F3—C10	1.3365 (16)	C8—C11	1.506 (2)
F4—C11	1.3440 (17)	C12—C13	1.5447 (18)
F5—C11	1.3376 (18)	C12—H12	1.0000
F6—C11	1.3372 (19)	C13—C17	1.5330 (19)
O1—C12	1.4159 (15)	C13—H13	1.0000
O1—H1O	0.841 (9)	C14—C15	1.522 (2)
O2—C18	1.2389 (18)	C14—H14A	0.9900
N1—C1	1.3092 (18)	C14—H14B	0.9900
N1—C9	1.3676 (17)	C15—C16	1.522 (2)
N2—C18	1.3489 (18)	C15—H15A	0.9900
N2—C14	1.4736 (18)	C15—H15B	0.9900
N2—C13	1.4833 (17)	C16—C17	1.527 (2)
C1—C2	1.4099 (18)	C16—H16A	0.9900
C1—C10	1.5155 (19)	C16—H16B	0.9900
C2—C3	1.3731 (19)	C17—H17A	0.9900
C2—H2	0.9500	C17—H17B	0.9900
C3—C4	1.4277 (18)	C18—C19	1.508 (2)
C3—C12	1.5284 (18)	C19—H19A	0.9800
C4—C5	1.4253 (18)	C19—H19B	0.9800
C4—C9	1.4233 (19)	C19—H19C	0.9800
C5—C6	1.365 (2)	O3—C20	1.417 (2)
C5—H5	0.9500	O3—H3O	0.844 (10)
C6—C7	1.410 (2)	C20—H20A	0.9800
C6—H6	0.9500	C20—H20B	0.9800
C7—C8	1.369 (2)	C20—H20C	0.9800
			
C12—O1—H1O	107.1 (13)	C3—C12—H12	107.9
C1—N1—C9	116.47 (12)	C13—C12—H12	107.9
C18—N2—C14	123.62 (12)	N2—C13—C17	111.09 (11)
C18—N2—C13	117.51 (11)	N2—C13—C12	109.90 (11)
C14—N2—C13	118.47 (11)	C17—C13—C12	115.71 (11)
N1—C1—C2	125.94 (12)	N2—C13—H13	106.5
N1—C1—C10	115.30 (12)	C17—C13—H13	106.5
C2—C1—C10	118.58 (12)	C12—C13—H13	106.5
C3—C2—C1	118.68 (12)	N2—C14—C15	111.64 (12)
C3—C2—H2	120.7	N2—C14—H14A	109.3
C1—C2—H2	120.7	C15—C14—H14A	109.3
C2—C3—C4	117.93 (12)	N2—C14—H14B	109.3
C2—C3—C12	120.24 (12)	C15—C14—H14B	109.3
C4—C3—C12	121.76 (12)	H14A—C14—H14B	108.0
C5—C4—C9	118.50 (12)	C16—C15—C14	110.70 (12)
C5—C4—C3	123.12 (12)	C16—C15—H15A	109.5
C9—C4—C3	118.37 (12)	C14—C15—H15A	109.5
C6—C5—C4	120.85 (13)	C16—C15—H15B	109.5
C6—C5—H5	119.6	C14—C15—H15B	109.5
C4—C5—H5	119.6	H15A—C15—H15B	108.1
C5—C6—C7	120.69 (13)	C15—C16—C17	109.83 (12)
C5—C6—H6	119.7	C15—C16—H16A	109.7
C7—C6—H6	119.7	C17—C16—H16A	109.7
C8—C7—C6	120.23 (13)	C15—C16—H16B	109.7
C8—C7—H7	119.9	C17—C16—H16B	109.7
C6—C7—H7	119.9	H16A—C16—H16B	108.2
C7—C8—C9	120.65 (13)	C16—C17—C13	115.50 (12)
C7—C8—C11	119.37 (12)	C16—C17—H17A	108.4
C9—C8—C11	119.98 (12)	C13—C17—H17A	108.4
N1—C9—C4	122.62 (12)	C16—C17—H17B	108.4
N1—C9—C8	118.31 (12)	C13—C17—H17B	108.4
C4—C9—C8	119.07 (12)	H17A—C17—H17B	107.5
F2—C10—F3	107.34 (11)	O2—C18—N2	120.45 (13)
F2—C10—F1	106.85 (11)	O2—C18—C19	119.45 (13)
F3—C10—F1	106.54 (11)	N2—C18—C19	120.07 (13)
F2—C10—C1	112.72 (11)	C18—C19—H19A	109.5
F3—C10—C1	113.03 (11)	C18—C19—H19B	109.5
F1—C10—C1	109.98 (11)	H19A—C19—H19B	109.5
F6—C11—F5	107.04 (13)	C18—C19—H19C	109.5
F6—C11—F4	106.43 (12)	H19A—C19—H19C	109.5
F5—C11—F4	106.05 (12)	H19B—C19—H19C	109.5
F6—C11—C8	112.55 (12)	C20—O3—H3O	107.1 (15)
F5—C11—C8	112.88 (12)	O3—C20—H20A	109.5
F4—C11—C8	111.45 (12)	O3—C20—H20B	109.5
O1—C12—C3	111.69 (10)	H20A—C20—H20B	109.5
O1—C12—C13	109.10 (10)	O3—C20—H20C	109.5
C3—C12—C13	112.24 (11)	H20A—C20—H20C	109.5
O1—C12—H12	107.9	H20B—C20—H20C	109.5
			
C9—N1—C1—C2	0.4 (2)	C2—C1—C10—F1	82.00 (15)
C9—N1—C1—C10	175.34 (11)	C7—C8—C11—F6	114.64 (15)
N1—C1—C2—C3	−0.1 (2)	C9—C8—C11—F6	−65.36 (17)
C10—C1—C2—C3	−174.91 (12)	C7—C8—C11—F5	−124.06 (15)
C1—C2—C3—C4	−0.56 (19)	C9—C8—C11—F5	55.94 (18)
C1—C2—C3—C12	176.59 (12)	C7—C8—C11—F4	−4.8 (2)
C2—C3—C4—C5	179.96 (13)	C9—C8—C11—F4	175.16 (13)
C12—C3—C4—C5	2.86 (19)	C2—C3—C12—O1	−16.35 (17)
C2—C3—C4—C9	0.88 (18)	C4—C3—C12—O1	160.69 (11)
C12—C3—C4—C9	−176.22 (11)	C2—C3—C12—C13	106.55 (14)
C9—C4—C5—C6	−0.2 (2)	C4—C3—C12—C13	−76.41 (15)
C3—C4—C5—C6	−179.28 (13)	C18—N2—C13—C17	144.40 (12)
C4—C5—C6—C7	0.9 (2)	C14—N2—C13—C17	−42.57 (16)
C5—C6—C7—C8	−0.7 (2)	C18—N2—C13—C12	−86.24 (14)
C6—C7—C8—C9	−0.2 (2)	C14—N2—C13—C12	86.79 (14)
C6—C7—C8—C11	179.85 (14)	O1—C12—C13—N2	−72.10 (13)
C1—N1—C9—C4	0.01 (19)	C3—C12—C13—N2	163.56 (10)
C1—N1—C9—C8	−179.10 (12)	O1—C12—C13—C17	54.71 (15)
C5—C4—C9—N1	−179.75 (12)	C3—C12—C13—C17	−69.64 (15)
C3—C4—C9—N1	−0.63 (19)	C18—N2—C14—C15	−137.78 (14)
C5—C4—C9—C8	−0.65 (19)	C13—N2—C14—C15	49.65 (16)
C3—C4—C9—C8	178.48 (12)	N2—C14—C15—C16	−55.41 (17)
C7—C8—C9—N1	179.97 (13)	C14—C15—C16—C17	56.65 (17)
C11—C8—C9—N1	0.0 (2)	C15—C16—C17—C13	−51.98 (16)
C7—C8—C9—C4	0.8 (2)	N2—C13—C17—C16	43.46 (16)
C11—C8—C9—C4	−179.18 (12)	C12—C13—C17—C16	−82.74 (15)
N1—C1—C10—F2	147.50 (12)	C14—N2—C18—O2	−174.09 (13)
C2—C1—C10—F2	−37.12 (17)	C13—N2—C18—O2	−1.46 (19)
N1—C1—C10—F3	25.55 (17)	C14—N2—C18—C19	7.7 (2)
C2—C1—C10—F3	−159.07 (12)	C13—N2—C18—C19	−179.67 (12)
N1—C1—C10—F1	−93.38 (14)		

### Hydrogen-bond geometry (Å, °)

**Table:**

*D*—H···*A*	*D*—H	H···*A*	*D*···*A*	*D*—H···*A*
O1—H1o···O3^i^	0.84 (2)	1.87 (2)	2.7121 (18)	177 (2)
O3—H3o···O2^ii^	0.845 (16)	1.834 (16)	2.6667 (17)	168 (2)
C7—H7···O1^iii^	0.95	2.49	3.3280 (18)	147
C17—H17a···F6^iv^	0.99	2.51	3.3123 (17)	138

Symmetry codes: (i) −*x*+1, −*y*+1, −*z*+1; (ii) *x*+1, *y*+1, *z*; (iii) *x*−1, *y*, *z*; (iv) −*x*, −*y*, −*z*+2.
